# Nicotine Enhances Interspecies Relationship between* Streptococcus mutans* and* Candida albicans*

**DOI:** 10.1155/2017/7953920

**Published:** 2017-02-09

**Authors:** Shiyu Liu, Wei Qiu, Keke Zhang, Xuedong Zhou, Biao Ren, Jinzhi He, Xin Xu, Lei Cheng, Mingyun Li

**Affiliations:** ^1^State Key Laboratory of Oral Diseases, Sichuan University, Chengdu, China; ^2^Department of Operative Dentistry and Endodontics, West China Hospital of Stomatology, Sichuan University, Chengdu, China

## Abstract

*Streptococcus mutans* and* Candida albicans* are common microorganisms in the human oral cavity. The synergistic relationship between these two species has been deeply explored in many studies. In the present study, the effect of alkaloid nicotine on the interspecies between* S. mutans* and* C. albicans* is explored. We developed a dual-species biofilm model and studied biofilm biomass, biofilm structure, synthesis of extracellular polysaccharides (EPS), and expression of glucosyltransferases (Gtfs). Biofilm formation and bacterial and fungal cell numbers in dual-species biofilms increased in the presence of nicotine. More* C. albicans* cells were present in the dual-species biofilms in the nicotine-treated groups as determined by scanning electron microscopy. The synthesis of EPS was increased by 1 mg/ml of nicotine as detected by confocal laser scanning microscopy. The result of qRT-PCR showed* gtfs* expression was upregulated when 1 mg/ml of nicotine was used. We speculate that nicotine promoted the growth of* S. mutans*, and more* S. mutans* cells attracted more* C. albicans* cells due to the interaction between two species. Since* S. mutans* and* C. albicans *are putative pathogens for dental caries, the enhancement of the synergistic relationship by nicotine may contribute to caries development in smokers.

## 1. Introduction

With an abundant supply of nutrients and diverse ecological niches, the human mouth is undoubtedly a good habitat for numerous microorganisms [1]. Over the past few decades, more than 700 different common oral species have been identified [2], which are part of the complex microbiota present in the human body.* Streptococcus mutans* is a common bacterial species residing in the oral cavity, especially in multispecies biofilms on the surfaces of teeth. It is an aerotolerant anaerobic bacterium that can ferment sugars and produce large quantities of glucans as well as acids, initiating demineralization and promoting the development of dental caries. Thus,* S. mutans* is one of the major cariogenic microorganisms in the oral cavity [3].

It has been estimated that 80% of human infections result from pathogenic biofilms [4]. Biofilm formation in the oral cavity leads to anaerobic as well as acidic conditions and both are relevant for the development of dental caries [5]. The capacity of* S. mutans* to form biofilms contributes to its cariogenicity. However, it has been reported that the ability of* S. mutans* to produce insoluble extracellular polysaccharide (EPS) through glucosyltransferases (Gtfs) plays a key role in cariogenic virulence [6]. EPS is the prime building block of dental biofilms and can promote* S. mutans *colonization on tooth surfaces, as well as attracting other microorganisms to form dental plaque. Consequently, a structured community or matrix is formed [7]. The EPS-rich matrix is a diffusion-limiting barrier, creating acidic microenvironments within the biofilms and resulting in the demineralization of dental hard tissues [8]. Several studies have indicated a high prevalence of* S. mutans *in dental plaques where the fungal pathogen* Candida albicans* resides, suggesting that these two species may interact [9, 10].

*C. albicans *is the most common human fungal pathogen and is normally harmless [11]. However, it would become opportunistically pathogenic when host has impaired immune function and is responsible for mucosal infections such as the vaginitis in women and oral-pharyngeal thrush in AIDS patients [12, 13].* C. albicans *is also a cariogenic microbe since it adheres to dental surfaces, forms biofilms, and produces acids [14, 15]. Recent investigations have indicated that* C. albicans *has been frequently found in early childhood caries (ECC) [16, 17]. Clinical studies have revealed that* S. mutans* and* C. albicans* are found together in dental plaques from toddlers with ECC [18, 19], suggesting that the interaction between these two species may mediate cariogenic development.

Autoagglutination between* C. albicans *and* S. mutans* has been observed [20] and extracellular materials were seen between* C. albicans *and* S. mutans *cells by scanning electron microscopy, suggesting that glucans play an important role in the development of dual-species biofilms [21]. These* C. albicans*/*S. mutans *biofilms reached higher biomass and cell numbers than single-species biofilms, while* S. mutans* EPS production was strongly suppressed [22]. An in vivo study also revealed a dramatic increase in the severity of smooth-surface lesions in the dually infected rats compared with singly infected rats [23].

Tobacco smoking has a documented impact on human health and in recent years many studies have found that smoking is closely associated with dental caries [24–27]. Higher scores of decayed, missing, or filled teeth (DMFT) were detected in Swedish smokers [28]. Nicotine is the most abundant alkaloid present in the cigarette. Interestingly, nicotine promotes growth, metabolic activity, and acid production in* S. mutans *[29, 30]. In addition, increased EPS synthesis and cell aggregation and higher overall lactate dehydrogenase activity of* S. mutans* were observed when nicotine was present [31].* C. albicans* has been found to have increased prevalence on the tongue of systemically healthy young smokers [32]. However, the association between nicotine and* C. albicans *has only been minimally investigated. Although there have been many studies focusing on the relationship between* S. mutans* and* C. albicans*, there have not been any reports concerning the effect of nicotine on their interspecies relationship. Considering that nicotine facilitates the growth of* S. mutans*, we hypothesize that nicotine may modulate the interspecies relationship between* S. mutans* and* C. albicans*. Since biofilms are the main pathogenic factor of oral microorganisms, we developed a dual-species biofilm model and studied the biofilms biomass, structures, EPS synthesis, and* gtfs* gene expression affected by physiologically relevant concentrations of nicotine.

## 2. Materials and Methods

### 2.1. Chemicals and Bacterial and Fungal Strains and Growth Conditions

Nicotine (>99% (GC), liquid) was purchased from Sigma-Aldrich (St Louis, MO, USA).* S. mutans* strain UA159 (ATCC 700610) and* C. albicans* strain SC5314 (ATCC 10691) were used in the present study. Precultures of* S. mutans* were grown in brain-heart infusion (BHI) medium at 37°C anaerobically with 5% CO_2_ [33]. Precultures of* C. albicans *were grown in YPD medium containing 1% yeast extract, 2% peptone, and 2% D-glucose at 37°C anaerobically with 5% CO_2_ [34]. YNBB (0.67% YNB, 75 mM Na_2_HPO_4_-NaH_2_PO_4_, 2.5 mM N-acetylglucosamine, 0.2% casamino acids, and 0.5% sucrose) was used to support the growth of* S. mutans *and* C. albicans *as well as biofilm formation [22]. The concentration of* S. mutans* was adjusted to 2 × 10^6^ colony-forming units (CFU)/ml and* C. albicans *to 2 × 10^4^ CFU/ml [23].

### 2.2. Biofilm Formation

Precultures of* S. mutans* and* C. albicans* from single colonies were incubated overnight and adjusted to a concentration of 2 × 10^7^ CFU/ml (*S. mutans*) and 2 × 10^5^ CFU/ml (*C. albicans*). Equal volumes of each strain (20 ul) and 160 ul YNBB medium were incubated into 96-well microtiter plates for the formation of dual-species biofilms. Suspensions (20 ul) of one strain only (*S. mutans *or* C. albicans*) and 180 ul YNBB medium were incubated into 96-well microtiter plates to form single-species biofilms. Equal volumes of each strain (200 ul) and 1.6 ml YNBB medium were also incubated in 24-well microtiter plates for dual-species biofilm formation. The plates were incubated at 37°C anaerobically with 5% CO_2_ for 24 h.

### 2.3. Minimum Inhibitory Concentration (MIC)

The twofold dilution method was used to determine the MIC of nicotine for* S. mutans* and* C. albicans* [29]. Overnight cultures of* S. mutans* (2 × 10^6^ CFU/ml) and* C. albicans* (2 × 10^4^ CFU/ml) were treated with 0, 1, 2, 4, 8, 16, and 32 mg/ml of nicotine in 96-well microtiter plates at 37°C anaerobically with 5% CO_2_ for 24 h. The optical density (OD) of each well was measured at 595 nm in a spectrophotometer.

### 2.4. Biofilm Biomass Assay by Crystal Violet Staining

After being incubated in 96-well microtiter plate for 24 h, the biofilm was gently washed with phosphate buffered saline (PBS), fixed with 95% methanol, washed with PBS, stained with 0.5% crystal violet for 30 min, and then washed with PBS. The crystal violet was extracted with 200 ul of 100% ethanol and the extract was read at 600 nm in a spectrophotometer [29].

### 2.5. Quantification of Biofilm Biomass Affected by Nicotine (Colony-Forming Unit Counts, CFU)

After incubation in 96-well microtiter plate for 24 h, the biofilms were gently washed with PBS to remove planktonic cells. The biofilms were then scraped off from the bottom of each well in 96-well microtiter plate and mixed by vortexing with 200 ul of PBS. The biofilm suspension was diluted 1 : 10^4^ (for counting* C. albicans*) and 1 : 10^6^ (for counting* S. mutans*) with PBS.* C. albicans* was incubated on YPD solid medium at 37°C aerobically and* S. mutans* was incubated on BHI solid medium at 37°C anaerobically with 5% CO_2_ for 48 h. Colonies were counted following incubation [35, 36].

### 2.6. Morphology of Mixed Biofilms by Scanning Electron Microscopy (SEM)

After incubation in 24-well microtiter plates for 24 h, the biofilms were gently washed with PBS, fixed with 2.5% glutaraldehyde overnight at 4°C, and washed with PBS. The fixed biofilms were then dehydrated by a series of ethanol rinses (30, 50, 70, 80, 85, 90, and 95%), immersed for 10 min in 100% ethanol, and dried in a desiccator [29]. After sputter coating with gold-palladium, samples were analyzed in a scanning electron microscope at 2000x, 5000x, and 10000x magnification.

### 2.7. Confocal Laser Scanning Microscopy (CLSM) of EPS in Mixed Biofilms

Dual-species biofilms were grown in YNBB with 1 mg/ml nicotine and 1 uM Alexa Fluor 647® red fluorescent dye labeling EPS in 24-well microtiter plates, protected from light. The control group was not treated with nicotine. After incubation for 24 h, biofilms were gently washed with PBS and incubated with 1 uM SYTO® 9 green fluorescent dye at 4°C for 20 min in the dark. Biofilms were then washed with PBS and dried. ProLong gold antifade reagent was added to the biofilms and images were obtained by CLSM [37]. Image-Pro Plus was used to quantify the fluorescence levels.

### 2.8. Quantitative Real Time RT-PCR Analysis of* S. mutans *and* C. albicans *Specific Genes in Mixed Biofilm

Dual-species biofilms were grown in the YNBB medium with 1 mg/ml of nicotine for 24 h. The control group was not treated with nicotine. The RNA isolation, purification, and reverse transcription of cDNA were performed similarly to those described in previous studies [38, 39]. Fast SYBR Green Master Mix and appropriate primers [*S. mutans* 16S rRNA,* gtfB*, and* gtfC*,* gtfD*, 0.375 mM, Table 1 [40]] as well as 2 ug of cDNA were used for quantitative PCR. The qPCR was performed on an ABI Prism 7000 system. 2^−ΔΔCt^ method was used to calculate* S. mutans gtfs* gene expression fold change values [41].

### 2.9. Statistical Analysis

Each experiment was independently repeated at least three times. One-way Analysis of Variance (ANOVA) was used to analyze the crystal violet staining, viable cell counts, and qPCR. The data were analyzed by SPSS 21.0 software. *P* < 0.05 was considered to be statistically significant.

## 3. Results

### 3.1. MIC

The MIC of nicotine against* S. mutans* was 16 mg/ml. The MIC of nicotine against* C. albicans* was 8 mg/ml. Considering the nicotine concentrations in human oral cavity (see the Discussion) and the MIC of nicotine against* S. mutans* and* C. albicans*, we used 1, 2, and 4 mg/ml of nicotine in the present study.

### 3.2. Nicotine Increased Biomass of Single* S. mutans* Biofilms and Dual-Species Biofilms

**Figure 1:**
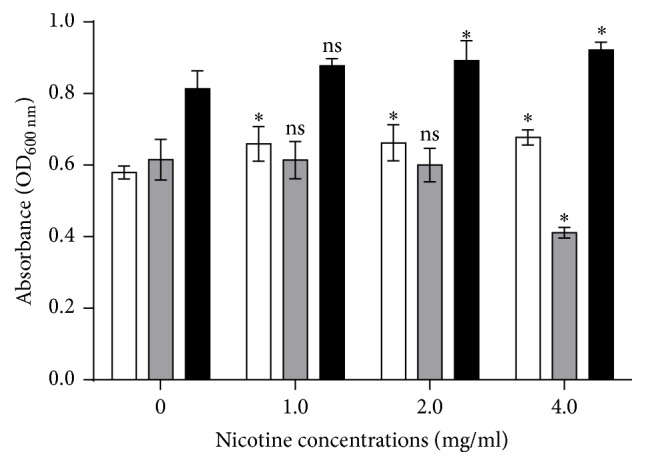
Biofilm biomass of single-species and dual-species biofilms at varying nicotine concentrations (0, 1, 2, and 4 mg/ml) at OD_600 nm_. The white bars indicate* S. mutans*, the grey bars indicate* C. albicans,* and the black bars indicate dual-species of* S. mutans* and* C. albicans*. Asterisks indicate the statistical differences compared to the 0 mg/ml nicotine control. The error bars indicate the standard deviation (SD). ^**∗**^*P* < 0.05 and ns: no significance.

(Figure 1) Single* S. mutans* biofilm biomass and dual-species biofilm biomass slightly increased in the presence of nicotine, 1.17-fold and 1.13-fold, respectively. For single* C. albicans *biofilms, however, lower nicotine concentrations had no obvious effect (1 and 2 mg/ml) on biofilm formation, while higher nicotine concentrations (4 mg/ml) inhibited biofilm formation.

### 3.3. Biofilm Colony Numbers Were Increased by Nicotine

**Figure 2:**
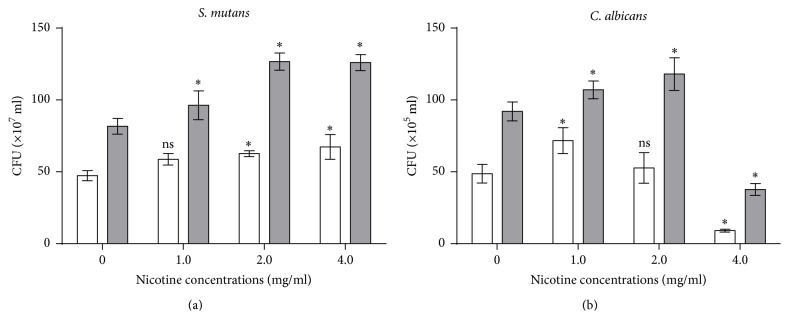
The number of colony-forming units (CFU) per biofilm at different nicotine concentrations (0, 1, 2, and 4 mg/ml). The white bars indicate single-species biofilms, and the grey bars indicate dual-species biofilms. Asterisks indicate the statistical differences compared to the 0 mg/ml nicotine control. The error bars indicate the standard deviation (SD). ^**∗**^*P* < 0.05 and ns: no significance.

To detect the respective cell number changes of* S. mutans* and* C. albicans* affected by nicotine in the dual-species biofilms, we calculated the biofilm colony numbers (Figure 2). For single* S. mutans* biofilms, CFU increased in nicotine-treated groups. For single* C. albicans* biofilms, CFU increased at 1 mg/ml of nicotine and decreased at 4 mg/ml of nicotine, with no statistical difference seen at 2 mg/ml of nicotine. The number of bacterial cells increased in the presence of nicotine in dual-species biofilms. Similarly, the number of fungal cells was increased in the presence of 1 and 2 mg/ml of nicotine but decreased at a nicotine concentration of 4 mg/ml in dual-species biofilms.

### 3.4. Nicotine Promoted* C. albicans* Attachment to* S. mutans*

**Figure 3:**
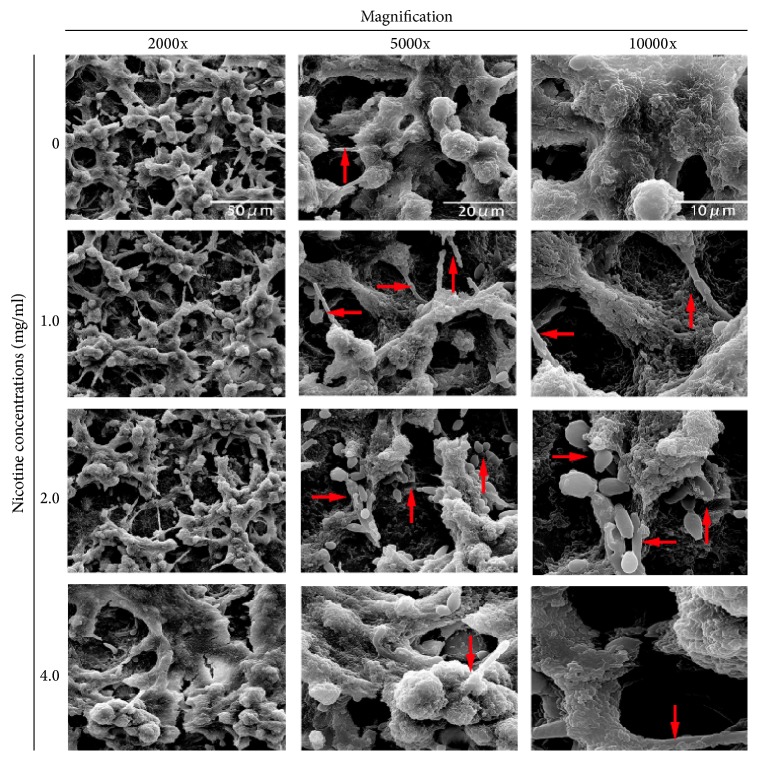
Morphology of dual-species biofilms treated with 0, 1, 2, and 4 mg/ml of nicotine for 24 h in YNBB broth. Magnification was 2000x, 5000x, and 10000x, respectively, for each concentration. The red arrows highlight* C. albicans* cells in yeast or hyphal forms.

Scanning electron micrographs display the distribution of* S. mutans* and* C. albicans* cells inside the dual-species biofilms (Figure 3).* C. albicans* cells were surrounded by* S. mutans* cells in dual-species biofilms. There were no obvious differences in the biofilm density between different nicotine concentration groups. However, there were differences in* C. albicans* attachment to dual-species biofilms between diverse nicotine concentration groups. In the absence of nicotine, only a few* C. albicans* cells were present in the coculture biofilms.* C. albicans *cells made up a greater proportion of the biofilms at nicotine concentrations of 1 and 2 mg/ml.

### 3.5. Nicotine Increased* S. mutans* Cell Numbers and EPS Production

**Figure 4:**
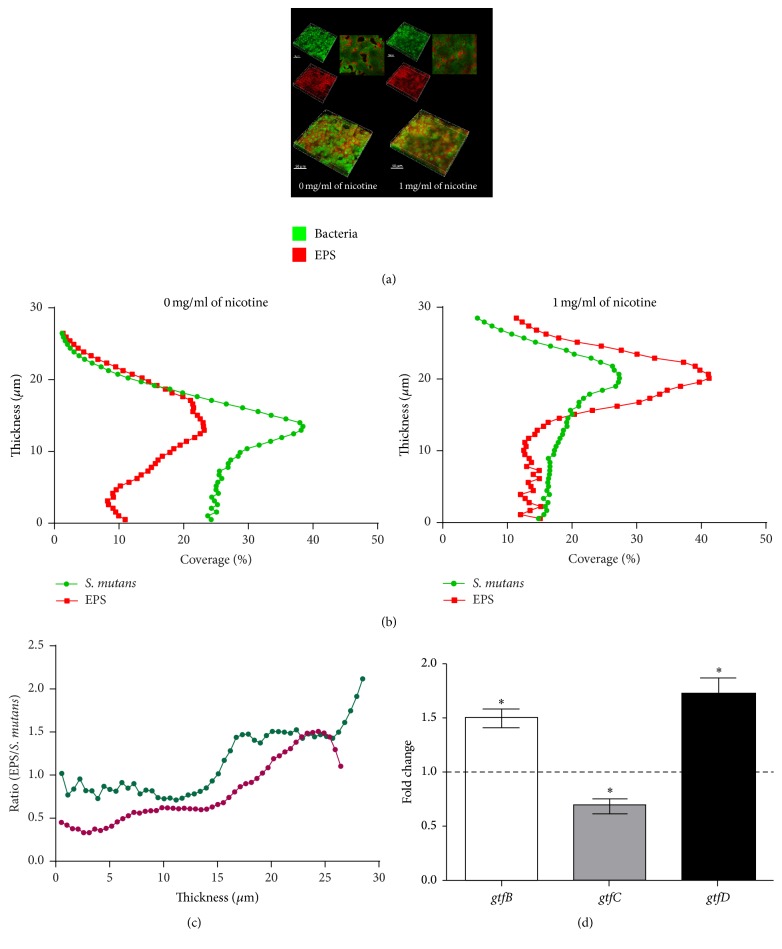
Confocal laser scanning microscopy images of dual-species biofilms. (a) A three-dimensional reconstruction of biofilms for 1 mg/ml nicotine-treated and the control group without nicotine. Reconstruction of the biofilm was performed with IMARIS 7.0.0. Bacterial cells were labeled green (SYTO 9), EPS was labeled red (Alexa Fluor 647), and red and green superimposed appear as yellow. Images were obtained at 60x magnification. (b) The distribution of EPS and bacteria in the reconstructed biofilm. (c) The ratio of EPS/*S. mutans*; the purple line is 0 mg/ml, and the green line is 1 mg/ml nicotine. (d) Expression of* S. mutans* EPS associated genes in dual-species biofilms treated with 1 mg/ml nicotine. Asterisks indicate the statistical differences compared with the 0 mg/ml nicotine control. The error bars indicate the standard deviation (SD). ^**∗**^*P* < 0.05.

The EPS play a key role in* S. mutans* cariogenic virulence since the EPS-matirx limits acids diffusion. Both* S. mutans* bacterial cell numbers and EPS production were increased by nicotine (1 mg/ml), as determined by CLSM images. According to the three-dimensional reconstruction images (Figure 4(a)), biofilms were more dense in the nicotine-treated groups. In the absence of nicotine, bacterial aggregates were sparse, while the aggregates became compact in the presence of 1 mg/ml of nicotine. The EPS around the bacterial cells was also more abundant with nicotine treatment. The data in Figure 4(b) showed the distribution of the biofilms. The ratio of EPS/*S. mutans* showed the capacity of* S. mutans* to produce polysaccharide (Figure 4(c)). This ratio increased at 1 mg/ml of nicotine.

### 3.6. Nicotine Influences Gene Expression in* S. mutans*

The Gtfs are the enzymes that catalyze the transformation of glucosyl groups and contribute to the synthesis of EPS by* S. mutans*. Expression of* gtfs* gene is closely associated with EPS synthesis. The effects of nicotine on* gtfs *gene expression are shown in Figure 4(d). The mRNA levels of bacterial* gtfB *and* gtfD* were increased 1.5- and 1.7-fold, respectively, at 1 mg/ml of nicotine. The mRNA level of bacterial* gtfC* decreased 0.70-fold (*P* < 0.05) in 1 mg/ml of nicotine.

## 4. Discussion

Bacterial-fungal interactions occur commonly in the human body and it has been shown that their interactions may influence the transition from a healthy state to a sick state within a specific host niche [42].* S. mutans* and* C. albicans* are typical bacteria and fungi in the oral microecosystem. They are found together in the oral environment and particularly in biofilms [9, 10].

It has been reported that the concentrations of nicotine in smokers' saliva range within 0.07–1.56 mg/ml [43], 0.096–1.6 mg/ml [44], or 0–1.33 mg/ml for light or medium smokers and 0–2.27 mg/ml for heavy smokers [45]. Another study measured a nicotine range of 0.367 to 2.5 mg/ml in stimulated saliva and 0.9 to 4.6 mg/ml in unstimulated saliva [46]. Considering the nicotine concentration ranges in saliva, we used 0, 1, 2, and 4 mg/ml of nicotine to get a physiologically relevant understanding of the effect of nicotine on the formation of single-species and dual-species biofilms.

For single* S. mutans* biofilms, there was a minor increase in biomass in the presence of nicotine. The increase was also seen in in the dual-species biofilms. The consistency between the increases in biofilm biomass between single* S. mutans* and dual-species biofilms could be explained by the promoting effect of nicotine on* S. mutans. *However, this does not take into account the role of* C. albicans* in dual-species biofilms. It should be noted that crystal violet staining of* C. albicans* biofilms is limited by the ability of the fungal cells to grow as both yeast and hyphal forms. Hyphae exhibit multicellular structures and have a larger biomass than yeast forms [23]. Therefore, in the present study, we also counted the CFU from the* S. mutans* and* C. albicans* single- and dual-species biofilms. The difference between crystal violet staining and viable cell counts for single* C. albicans* could be explained by the morphology changes in the different nicotine concentration groups. Interestingly dual-species biofilms displayed more* S. mutans* microcolonies than single species. This phenomenon might be induced by the presence of* C. albicans*. Synergistic interactions between the two species have been demonstrated in many other studies [21–23].* S. mutans* has been demonstrated to coadhere with* C. albicans* through EPS or GtfB synthesized by* S. mutans* [6, 22]. However, another factor (nicotine) was added in the present study. Here, we showed that nicotine strengthened the dual-species interactions. There were more bacterial and fungal cells with nicotine treatment. And this conclusion was supported by the SEM data. More* C. albicans* cells were seen in the biofilms at nicotine concentrations of 1 and 2 mg/ml. In high concentration of nicotine (4 mg/ml),* S. mutans* plays an essential role in modulating the competitive fitness of* C. albicans* by alleviating the inhibitory effect of nicotine, thus promoting the survival and persistence of* C. albicans* within the biofilms.

Considering that EPS is the main virulence factor for* S. mutans* cariogenicity and most studies have shown that the nicotine concentration in oral saliva is approximately 1 mg/ml, we used 1 mg/ml of nicotine to explore EPS synthesis and the expression of related genes in dual-species biofilms. From the three-dimensional reconstruction of the biofilm, both bacterial cells and EPS synthesis increased at 1 mg/ml nicotine. The 3-dimensional structure of the biofilm shows an overall image of EPS and bacterial cells in the biofilm; however, it does not show the distribution of EPS and bacterial cells in each layer. We calculated the coverage of* S. mutans* cells and EPS at each layer of the biofilm at each pixel site. The ratio of EPS/bacteria was increased in the 1 mg/ml nicotine group, indicating that increased EPS synthesis could be attributed to nicotine treatment. As mentioned previously, EPS is capable of attracting other microorganisms onto the dental plaque due to its ability to provide binding sites for cell attachment [6, 7]. Since there was more EPS present in the environment, bacterial and fungal cells were more likely to aggregate, resulting in higher biofilm mass. The compact biofilm creates an anoxic and acidic environment, leading to an imbalance between enamel demineralization and remineralization, leading to demineralization of the dental hard tissues. In addition, EPS also acts as a sugar supply that can be fermented to acids. As a consequence, nicotine may increase caries occurrence and promote caries development in smokers.

Gtfs are essential for* S. mutans* utilization of glucose and for EPS synthesis and are a contributing factor to biofilm formation and the development of caries. Three different Gtfs are expressed by* S. mutans*: GtfB, GtfC, and GtfD. They are, respectively, encoded by the genes* gtfB*,* gtfC*, and* gtfD*. It has been revealed that the soluble polysaccharide metabolite produced by GtfD serves as the primer for GtfB [7]. This could explain the similar trends in* gtfB* and* gtfD* expression in the nicotine-treated group. Both* gtfB* and* gtfD* expression were upregulated in 1 mg/ml of nicotine (*P* < 0.05, Figure 4(d)). GtfB and GtfC synthesize *α*-1,3-rich water-insoluble polysaccharide [47], and the lack of* gtfB* or* gtfC* disrupts* C. albicans* colonization of* S. mutans*-*C. albicans* biofilms [23]. However, it should be noted that the glucans synthesized by* S. mutans* GtfB are considered to be crucial for bacterial-fungal coadhesion [48]. GtfB binds to both yeast and hyphal form cell surfaces and still remains enzymatically active, further converting* C. albicans* into a de facto glucan producer [23]. Upregulated* gtfB* gene expression in 1 mg/ml of nicotine may be explained by the increased numbers of bacterial and fungal cells that required more EPS and GtfB to adhere to each other. One study revealed that* S. mutans* EPS production was strongly suppressed in dual-species biofilms [22]. However, the ratio of EPS/*S. mutans* and* gtfs* expression was elevated in the presence of 1 mg/ml nicotine in the present study. Compared with* C. albicans*, nicotine had a stronger influence on EPS synthesis by* S. mutans*.

**Figure 5:**
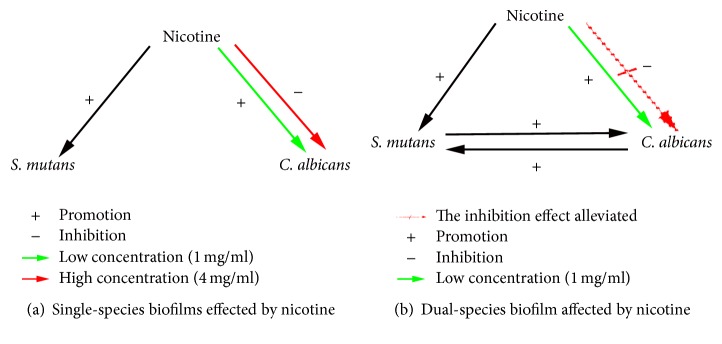
Relationship between nicotine,* S. mutans,* and* C. albicans*.

We have summarized the relationship between* S. mutans*,* C. albicans,* and nicotine (Figure 5). Nicotine promoted the growth of* S. mutans* both in pure cultures and in cocultures. A low concentration (1 mg/ml) of nicotine promoted the growth of* C. albicans* in pure cultures and in cocultures, and a high concentration (4 mg/ml) of nicotine inhibited the growth of* C. albicans* in pure cultures and in cocultures. However, the inhibitory effect was alleviated in coculture medium as more* C. albicans* microcolonies were present in the dual-species biofilms compared to the single-species biofilms (37.67 ± 4.16 CFU versus 9 ± 1.0 CFU, Figure 2). This suggests that there is a genuine interaction between the two species and they promote the growth of each other.

In summary, we propose that nicotine promotes biofilm formation and coadhesion of* S. mutans* and* C. albicans* in dual-species biofilms. Furthermore, nicotine increases EPS synthesis by* S. mutans* and 1 mg/ml of nicotine stimulates* S. mutans gtfs* (*gtfB* and* gftD*) expression. As* C. albicans* and* S. mutans* are putative pathogens for dental caries, the enhancement of nicotine on the synergistic relationship between* S. mutans* and* C. albicans* may contribute to caries development in smokers. However, this assumption requires further work in order to be confirmed.

## Figures and Tables

**Table 1:** Specific primers used for qPCR.

Primers	Sequences
16S rRNA	
F	5′-AGCGTTGTCCGGATTTATTG-3′
R	5′-CTACGCATTTCACCGCTACA-3′
gtfB	
F	5′-CACTATCGGCGGTTACGAAT-3′
R	5′-CAATTTGGAGCAAGTCAGCA-3′
gtfC	
F	5′-GATGCTGCAAACTTCGAACA-3′
R	5′-TATTGACGCTGCGTTTCTTG-3′
gtfD	
F	5′-TTGACGGTGTTCGTGTTGAT-3′
R	5′-AAAGCGATAGGCGCAGTTTA-3′
