# Corrigendum to “Nicotine Enhances Interspecies Relationship between* Streptococcus mutans* and* Candida albicans*”

**DOI:** 10.1155/2017/5803246

**Published:** 2017-09-20

**Authors:** Shiyu Liu, Wei Qiu, Keke Zhang, Xuedong Zhou, Biao Ren, Jinzhi He, Xin Xu, Lei Cheng, Mingyun Li

**Affiliations:** ^1^State Key Laboratory of Oral Diseases, Sichuan University, Chengdu, China; ^2^Department of Operative Dentistry and Endodontics, West China Hospital of Stomatology, Sichuan University, Chengdu, China

In the article titled “Nicotine Enhances Interspecies Relationship between* Streptococcus mutans* and* Candida albicans*” [1], an acknowledgment should be added as follows.

This study was supported by the National Natural Science Foundation of China (81400501 to Mingyun Li and 81430011 to Xuedong Zhou), the International Science and Technology Cooperation Program of China (2014DFE30180 to Xuedong Zhou), and the Special Fund of State Key Laboratory of Oral Diseases, Sichuan University (SKLOD201525 to Mingyun Li).
